# Codesigning a Nurse-Led, Large Language Model-Empowered Agent to Increase Hepatitis B Screening and Vaccination for Inclusion Health Populations: A Research Protocol

**DOI:** 10.3390/nursrep16020074

**Published:** 2026-02-19

**Authors:** Caixia Li, Wei Xia, Zheng Zhu, Marques Shek Nam Ng, Xia Fu

**Affiliations:** 1 The Department of Nursing, The Eighth Affiliated Hospital, Sun Yat-sen University, 5/F The Administration Building, Shenzhen 518033, China; licx23@mail.sysu.edu.cn; 2 The School of Nursing, Sun Yat-sen University, Guangzhou 510080, China; xiaw23@mail.sysu.edu.cn; 3 The School of Nursing, Fudan University, Shanghai 200032, China; zhengzhu@fudan.edu.cn; 4 The Nethersole School of Nursing, The Chinese University of Hong Kong, Hong Kong 999077, China; marquesng@cuhk.edu.hk

**Keywords:** agent, double diamond model, hepatitis B, inclusion health populations, large language model, nurse-led intervention, nursing, screening, vaccination

## Abstract

**Background/Objectives:** We aim to codesign and test a nurse-led, large language model-empowered agent to increase hepatitis B screening and vaccination for inclusion health populations. **Methods**: This study employs a double diamond model-guided codesign methodology. It includes four phases: (i) Discover: To identify intervention targets, a systematic review was undertaken that synthesized 51 factors influencing hepatitis B screening and vaccination among inclusion health populations. A qualitative study will later be conducted to further elucidate specific cultural barriers in the Chinese context. (ii) Define: To delineate effective intervention designs, two systematic reviews were performed, informing the integration of nurse-led intervention components (e.g., counseling, case management, and care coordination) and adaptation of a large language model to address identified intervention targets. (iii) Develop: To codesign an agent, hepatitis B prevention datasets will be constructed with subsequent model adaptations through fine-tuning and retrieval-augmented generation, as well as collaborations among diverse stakeholders. It will facilitate human–agent interactive consultation, intelligent case management, and care coordination, as well as collaborate with a nurse-led multidisciplinary team to manage hepatitis B screening, vaccination, and care linkage. (iv) Deliver: To evaluate and refine the agent, a mixed-methodology will be adopted, encompassing quantitative evaluation of model response, as well as qualitative evaluation of user experience, technical barriers, and potential benefits. **Discussion**: This intervention is expected to improve hepatitis B screening and vaccination rates among inclusion health populations, thereby enhancing diagnosis, immunity, and care linkage. It will establish a codesign framework for nursing-specific large language models, broadening the impact of nurses on preventive health equity.

## 1. Introduction

“*When the Great Way prevails, the world community is equally shared by all*.”——Confucius

This profound adage from the Analects of Confucius envisions a society in which resources and opportunities are equitably distributed [1]. In this vision, health equity advocates for a fair distribution of health resources and outcomes, ensuring equal opportunities to achieve optimal health outcomes for all [2]. Nonetheless, inequities continue to challenge health outcomes for inclusion health populations, particularly in the efforts to eliminate hepatitis B virus (HBV) due to their insufficient access to preventive health resources, including screening and vaccination services [3].

HBV, a major liver disease etiology, disproportionately affects inclusion health populations, including rural residents, migrant workers, and individuals of low socio-economic status [3]. Their high-risk behaviors, including tattooing, unprotected sex, and blood selling, results in elevated HBV exposure rate ranging from 10.1% to 31.8% [4,5]. The HBV prevalence in these populations, ranging from 4.4% to 39.2% [6], significantly exceeds that in the general population (3.3%) [7]. Nonetheless, a systematic review showed that less than 28% of inclusion health populations undergo HBV screening and fewer than 30% complete a three-dose HBV vaccination regimen [3]. Consequently, approximately 90% of HBV cases remain undiagnosed [8] and 86% of inclusion health populations remain unimmunized [9], perpetuating disease transmission, exacerbating advanced liver diseases, and threatening global targets for eliminating HBV by 2030 [10]. Ensuring equitable access to HBV screening and vaccination services is imperative.

As emphasized by the International Council of Nurses [11], nurses are professionally obligated to promote health equity. Nurse-led interventions, including health education, counseling, and case management, are pivotal in addressing cognitive, emotional, and social barriers to HBV screening and vaccination in inclusion health populations, including unawareness of HBV risks, knowledge deficits in HBV prevention, fear of HBV detection, and access problems [12,13]. The synthesized data revealed that nurse-led interventions resulted in a pooled HBV screening rate of 64% and significantly enhanced the likelihood of HBV vaccination by 2.61-fold [14]. However, the reliance of these interventions on in-person delivery strains the nursing workforce and hampers participation of inclusion health populations. Moreover, their non-tailored intervention materials fail to address individual-specific healthcare access barriers [12], potentially diluting intervention effectiveness [14]. Innovative strategies are required to enhance nurses’ efficacy in HBV prevention for inclusion health populations.

Large language model-empowered agents offer transformative solutions. These agents are advanced artificial intelligence systems, excelling in processing, understanding, and generating natural language to foster effective and personalized human–artificial intelligence interactions [15]. With extensive pre-training and billions of parameters, they can generate contextually relevant recommendations for preventing and managing diverse diseases, including chronic liver diseases [16,17]. They can also tailor information to individual needs via in-context learning and fine-tuning [15]. The tailored information has shown to enhance individuals’ knowledge of ailments, preventive measures, and self-management techniques [16], thereby facilitating behavioral changes in disease prevention and screening [18]. Furthermore, the smartphone accessibility of these agents breaks geographical and access barriers [19], which could facilitate an equitable access to HBV preventive health information for inclusion health populations. Their scalability also allows for simultaneous interactions with multiple individuals, reducing reliance on the nursing workforce [16]. In particular, the tool-use capability enables agents to leverage external tools to accomplish diverse tasks, including accessing medical databases, answering medical questions, and performing health counseling [15]. This versatility positions them as promising platforms for nurse-led interventions to enhance HBV prevention for inclusion health populations.

The double diamond model provides robust guidance to develop nurse-led, large language model-empowered agents. The model encompasses a dual diverging-converging process across four phases—discover, define, develop, and deliver—to explore problems, refine problems, create solutions, as well as test and improve the solutions iteratively [20]. It also emphasizes the engagement of multiple stakeholders to gain a deeper understanding of user needs and codesign more equitable solutions [21]. This approach has shown to develop inclusive user-centric technological interventions, facilitate preventive health behaviors (e.g., vaccination), and reduce health inequities for inclusion health populations [22,23]. Hence, guided by the double diamond model, this study aims to codesign and test a nurse-led, large language model-empowered agent to improve HBV screening and vaccination rates in inclusion health populations.

## 2. Background

Hepatitis B virus poses a substantial public health threat, and the target of its global elimination by 2030 remains challenging [24]. It affected approximately 254 million people in 2022 and causes 1.2 million new infections annually [24]. Data from 187 countries indicate a rise in HBV-related deaths from 555,000 in 2019 to 1.1 million in 2022, positioning HBV as one of the few communicable diseases with an increasing mortality rate [24]. HBV-induced cirrhosis, hepatocellular carcinoma, liver failure, and extrahepatic complications further exacerbate its burden [25]. With 79,700,000 HBV infections, China has the largest HBV burden, representing approximately 31.5% of the global infections in 2022 [24]. However, less than 60% of the people living with chronic HBV in China are aware of their infection status [26]. Globally, the awareness rate was even lower, at 13.4% by 2022 [7]. This has led to a low antiviral treatment rate among the infected individuals (2.6%), perpetuating ongoing HBV transmission and advanced liver diseases [7]. Preventive efforts, including HBV screening, should be strengthened to alleviate the HBV burden.

Inclusion health populations, including rural residents, migrant workers, and individuals of low socioeconomic status, experience a disproportionately high HBV burden. The seroprevalence of HBV infection among rural residents in China [27], Nigeria [28], and Camerron [29] ranged from 6.7% to 10.7%. Migrant workers also face elevated risks, with a high proportion (58.19%) undertaking high-risk behaviors, such as unprotected sexual behavior [30]. Their HBV seroprevalence rates have reached 8.1% in China [31], 11.4% in Thailand [32], and 17.24% in Indonesia [33]. A pooled rate of 21.05% was reported among sanitation workers with low socioeconomic status [34]. Despite this elevated risk, less than 27.5% of these individuals were screened for HBV [3]. For instance, a recent study of 320,000 individuals, including poor people, rural residents, uninsured individuals, and unhoused people, found that only 16.3% had previously screened for HBV [35]. Additionally, the pooled proportions of HBV vaccination (at least one dose) and prior immunization among inclusion health populations were only 37% [3] and 14.2% [9], respectively. Studies also highlighted their low rates of follow-up testing (43%), treatment (15.9%), and specialist care linkage (32%) after HBV diagnosis [3,36]. Consequently, approximately 30% of them had liver cirrhosis at initial disease presentation and experience a high HBV-induced liver disease burden [36]. However, targeted interventions are lacking and should be developed to ensure equitable access to HBV preventive services for inclusion health populations.

Nurse-led interventions, including health education, counseling, case management, and care coordination, have high potential to address the multifaced disparities hindering HBV prevention in inclusion health populations. First, these populations exhibit significant health knowledge deficits regarding HBV, its symptoms, consequences, screening, and vaccination [3]. For instance, approximately 32.8% of inclusion health individuals, including rural residents and sanitation workers, had never heard of HBV, 41% were unaware that HBV primarily affects the liver, and 55% were unaware of the availability of HBV screening tests [37,38]. Nurse-led health education and counseling have effectively mitigated these knowledge gaps by enhancing awareness of HBV transmission, diagnosis, risk-reduction behaviors, screening procedures, and vaccination schedules [13], thereby facilitating informed utilization of HBV screening and vaccination [14]. Second, inclusion health populations face multiple service access barriers, including HBV vaccine costs, a lack of health insurance, demanding work schedules, and transportation and appointment scheduling difficulties [3]. Nurse-led case management employs multifaced strategies, including telephone follow-ups, behavior reminders, and financial incentives, to overcome these barriers and has proven effective in improving access to HBV screening and vaccination among inclusion health populations, such as homeless adults [39]. Third, unstable living and working conditions often result in frequent relocations among these populations, disrupting care linkages post HBV screening [3]. Nurse-led care coordination ensures service continuity by integrating primary and specialist hepatology care, facilitating further vaccination, liver imaging, HBV treatment, and disease surveillance [12,13]. However, the dependency on nursing manpower coupled with the scarcity of the nursing workforce in inclusion health communities, have constrained the implementation of these nurse-led interventions [14,40], promoting further exploration of artificial intelligence-assisted strategies.

Since the release of ChatGPT (GPT-3.5 model, OpenAI, San Francisco, CA, USA) in November 2022, large language models have gradually been integrated into the nursing profession to enhance healthcare [41]. Large language model-empowered agents have significantly contributed to answering medical inquiries, delivering health education, and providing counseling for patients with chronic diseases, including diabetes, hypertension, asthma, and liver cirrhosis [16,17]. Their integration into healthcare systems also aids nurses in care coordination and case management, including appointment scheduling, disease surveillance, and generating personalized management plans, ultimately amplifying nursing efficiency and patient outcomes [16,41]. Despite their potentials in nursing care, agents based on general-purpose large language models are trained using public data, which may generate irrelevant, fabricated, or misleading information for a specific nursing domain [41]. Consequently, fine-tuning and retrieval-augmented generation are routinely employed adaptation strategies. Fine-tuning involves changing a model’s weight to enhance its performance on a specific task [15]. Retrieval-augmented generation, by incorporating external knowledge bases, such as clinical practice guidelines, has significantly enhanced the accuracy of model responses by approximately 2.89 times [16]. Hence, this study will leverage these strategies to firstly develop a specialized large language model-empowered agent equipping nurse-led interventions to enhance HBV screening and vaccination in inclusion health populations.

Codesign is a participatory research approach that actively engages users to ensure that intervention designs are meticulously aligned with their specific needs [23]. Through a combination of methodologies, such as focus group interviews, Delphi consultations, and surveys, the codesign process facilitates the involvement of clinicians, patients, and service users [21]. The study materials and delivery strategies developed through the process are perceived as more applicable and acceptable to inclusion health populations [22], thereby enhancing the relevance and effectiveness of interventions. The double diamond model, a design process framework, also places strong emphasis on stakeholder involvement in identifying problems and developing innovative solutions [20]. This model has been successfully used to guide the codesign process for preventive healthcare services tailored to inclusion health populations [22,23]. This study will be guided by the double diamond model to facilitate the collaborative engagement of stakeholders, including inclusion health populations, healthcare professionals, and engineers, in the codesign of a nurse-led, large language model-empowered agent.

The specific research questions of the current study are (i) What are the factors influencing HBV screening and vaccination among inclusion health populations? and (ii) Is a nurse-led, large language model-empowered agent feasible for improving HBV screening and vaccination uptake among inclusion health populations? Accordingly, the objectives of this study are to develop a double-diamond-guided, nurse-led, large language model-empowered agent and to test its feasibility in improving HBV screening and vaccination uptake within these populations. This study will contribute to equitable HBV preventive healthcare access and the micro-elimination of HBV among the most vulnerable cohorts. Furthermore, the codesign methodology will provide guidance for integrating large language models into nurse-led interventions to address healthcare disparities.

## 3. Materials and Methods

### 3.1. Design

**Figure 1:**
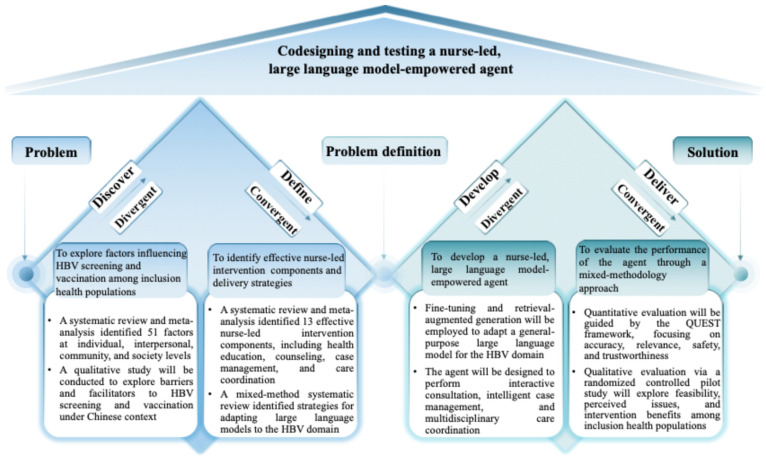
Codesigning and testing a nurse-led, large language model-empowered agent guided by the double diamond model. Abbreviations: HBV, hepatitis B virus; QUEST: quality of information, understanding and reasoning, expression style and persona, safety and harm, and trust and confidence.

This study is guided by the double diamond model [20], encompassing four phases (Figure 1): (i) Discover (in progress): to explore factors influencing HBV screening and vaccination among inclusion health populations to determine intervention targets; (ii) Define (completed): to identify effective nurse-led intervention components and delivery strategies targeting key influencing factors; (iii) Development (pending): to develop a nurse-led, large language model-empowered agent; (iv) Deliver (pending): to evaluate the performance of the agent through a mixed-methodology approach to iteratively refine it. The study protocol was registered on the Chinese Clinical Trial Registry (ChiCTR2500104184) on 12 June 2025. It was reported following the SPIRIT (Standard Protocol Items: Recommendations for the Intervention Trials) statement (Supplementary Material S1) [42].

### 3.2. Discover Phase

This phase aims to identify factors influencing HBV screening and vaccination among inclusion health populations through a mixed-method systematic review and a qualitative study. The review has been completed. It involved searching 11 databases and included 21 studies examining disparities in HBV screening and vaccination among four inclusion health groups, including sex workers, rural migrant workers, underserved immigrants, and individuals with a low socio-economic status [3]. It identified 51 influencing factors, guiding this study to focus on key modifiable factors as intervention targets: (i) health knowledge about HBV; (ii) health beliefs regarding HBV screening and vaccination, including fear of detecting HBV; and (iii) barriers to healthcare access, such as geographic mobility [3]. Given that most of the included studies originated from Western countries, their findings may not apply fully to the Chinese context, necessitating an exploratory qualitative study.

A qualitative study using an interpretative phenomenological approach [43] will be conducted to further explore specific cultural barriers and facilitators in the Chinese context. It will recruit participants who predominantly represent inclusion health populations in China, specifically targeting (i) rural residents (individuals holding a rural household registration), (ii) rural migrant workers, and (iii) individuals with low socioeconomic status, such as sanitation and construction workers. Eligible participants must be aged 18 to 65 years and demonstrate the cognitive capacity and communication proficiency required to provide informed consent and engage in the interview. Individuals will be excluded if they have a confirmed prior diagnosis of HBV infection, severe psychiatric disorders, malignancy, or end-stage diseases that could impede participation.

Participants will be recruited via purposive sampling from two university-affiliated hospitals and their community health centers, with the sample size determined by data saturation. The principal researcher will conduct semi-structured interviews using open-ended questions: (i) How much do you know about hepatitis B? (ii) How do you perceive hepatitis B prevention? (iii) What do you think about hepatitis B screening? (iv) How much do you know about the hepatitis B vaccine? (v) If you plan to undergo hepatitis B screening/vaccination, what difficulties would you face? Interviews will be audio-recorded, transcribed verbatim, and analyzed using interpretative phenomenological analysis [44]. These findings will further inform the intervention targets.

### 3.3. Define Phase

This phase aims to identify effective intervention components and delivery strategies that address the key factors influencing HBV screening and vaccination among inclusion health populations. For intervention components, a systematic review and meta-analysis was performed that examined 13 databases, included 16 studies, and identified 13 nursing components that have effectively improved HBV screening and vaccination among inclusion health and general populations [14]. It recommended this study to incorporate (i) health education, counseling, and decision support to improve health knowledge, health beliefs, and informed utilization of HBV screening and vaccination; (ii) case management to address barriers to HBV screening and vaccination; and (iii) care coordination and referral to promote specialist care linkage (including HBV treatments) post-HBV screening.

To clarify delivery strategies, a mixed-method systematic review was conducted [16]. By searching 11 databases and reviewing 20 included studies, it found that large language models (i) provided equitable access to health information through internet-enabled devices, particularly for inclusion health populations; (ii) enhanced health knowledge and beliefs in preventing and managing chronic diseases, including liver diseases; and (iii) facilitated preventive health behaviors [16]. These models could be used to assist in the delivery of nurse-led intervention components, such as health education and counseling. However, the review noted a 71% accuracy rate for responses from general-purpose large language models, with adaptation strategies (including retrieval augmented generation) improving the accuracy rate by approximately 2.89 times [16]. Hence, this study will apply the model adaptation strategies to enhance its response accuracy.

### 3.4. Development Phase

**Figure 2:**
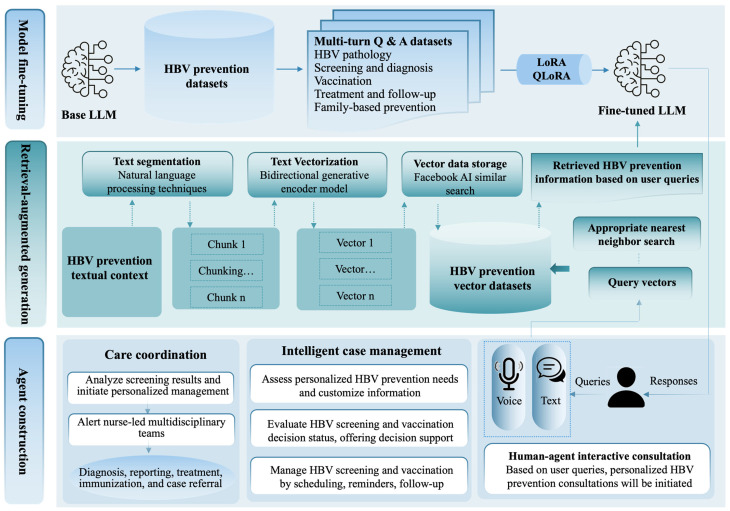
Development of the nurse-led, large language model-empowered agent (Phase III). Abbreviation: HBV, hepatitis B virus; LoRA, low-rank adaptation; QLoRA, quantized low-rank adaptation; LLM, large language model.

This phase aims to develop a large language model-empowered agent. It is grounded in (i) key modifiable factors influencing HBV screening and vaccination among inclusion health populations, as identified in the discover phase [3], (ii) effective nurse-led intervention components and delivery strategies leveraging large language models, as determined in the define phase [14,16], and (iii) established HBV prevention and management guidelines. The nurse-led, large language model-empowered agent will be developed with the following steps (Figure 2):

*Selection of a foundation model for the agent.* An optimal foundation model for the agent targeting HBV prevention will be selected from open-source large language models, such as ChatGLM3-6B (Zhipu AI Co., Ltd., Beijing, China). A standardized set of HBV-related question–answer pairs will be used to evaluate model performance in terms of text generation ability, inference speed, resource consumption, and coverage of HBV-specific knowledge. A model that effectively balances performances (meeting a minimum accuracy threshold of 60% on HBV specific questions) and efficiency for HBV prevention information will be selected for further fine-tuning. In addition, an optical character recognition model will be integrated to extract textual content from user-uploaded images (including HBV screening reports) to support downstream question-answering using the large language model. To address safety concerns, all uploaded reports and optical character recognition text will be processed transiently and purged immediately, thereby eliminating server storage and minimizing data retention risks. Furthermore, to enhance accessibility for users with limited literacy, a voice interaction system will be implemented. It will incorporate an automatic speech recognition model to transcribe user speech into text and a text-to-speech model to convert the generated responses into audio output, thereby enabling inclusive access to HBV prevention counseling.

*Compiling datasets regarding HBV prevention.* Guidelines, question–answer pairs, and tailored information will be used to construct datasets for HBV prevention. It will serve as a resource for model fine-tuning and can be incorporated into a retrieval-augmented generation framework to enhance the model’s knowledge of HBV prevention. This study will search HBV prevention and management guidelines from computerized decision systems (n = 2), guideline websites (n = 4), liver disease professional association websites (n = 4), and electronic databases (n = 10). Details of the search sources and keywords are presented in Supplementary Material S2 (Tables S1 and S2). Additionally, question–answer pairs regarding HBV published by open internet consultation records and authoritative organizations (e.g., China disease prevention and control centers) will also be collected. Moreover, tailored information will be created to address individual variations in HBV knowledge, health beliefs regarding HBV screening and vaccination, and barriers to healthcare access. This can be achieved by assessing individual needs using validated instruments and customizing messages based on the responses. An example of tailoring algorithms and information using messaging techniques is presented in Supplementary Material S2 (Table S3).

The datasets will be preprocessed. This includes removing invalid characters using regular expressions, standardizing medical terminologies with medical dictionaries (e.g., unifying hepatitis B virus, HBV, and hepatitis B), and assigning version identifiers for traceability. Text similarity algorithms will be applied to remove redundant information, while irrelevant or noisy content will be filtered out through manual review and natural language processing-based relevance assessment, minimizing the impact of hard negative samples.

*Model fine-tuning*. It aims to enable the model to support multi-turn question–answering in HBV prevention, covering five key areas: (i) HBV pathology (e.g., HBV transmission routes, symptoms, and disease progression), (ii) HBV screening and diagnosis, (iii) HBV vaccination, (iv) HBV treatment and follow-up care, and (v) family-based HBV prevention strategies. Relevant datasets will be formatted into multi-turn question–answering samples using instruction, input, and output structures. The model will be fine-tuned using a combination of low-rank adaptation and quantized low-rank adaptation techniques. The former freezes the original model weights and introduces low-rank adapter layers into the pre-trained architecture, significantly reducing the number of trained parameters while preserving the model’s original knowledge, as well as lowering the computational and memory costs [45]. Quantized low-rank adaptation integrates 4-bit normal float quantization, which reduces memory usage and enables fine-tuning of devices with constrained computational capabilities [45].

*Enhancing the fine-tuned large language model using retrieval-augmented generation.* The fine-tuned model will be further enhanced using retrieval-augmented generation, which integrates external HBV prevention knowledge through indexing, retrieval, and generation [46]. In the indexing phase, the HBV prevention datasets will be processed and stored in a vector database using Facebook AI similarity search. The textual context will be segmented into semantically meaningful chunks using natural language processing techniques to preserve domain-specific medical terminology. These chunks will then be embedded using a BAAI general embedding model and stored as fixed-dimensional vectors for efficient similarity computation. During the retrieval phase, user queries will be encoded using the same embedding model and mapped onto the vector space. An appropriate nearest neighbor search will be applied to retrieve the most relevant text vectors based on the queries. A reranking mechanism that considers keyword weighting, temporal recency, and source authority will be introduced to refine the retrieved results. Finally, the retrieved information will be paired with user queries and fed into the large language model to improve the relevance and accuracy of its generations regarding HBV prevention.

*Constructing the large language model-empowered agent.* A front-end application will be developed based on the WeChat Mini Program platform to support core user functionalities, including registration, login, document upload (e.g., HBV screening reports), and individualized HBV prevention needs assessment. It will integrate the adapted large language model using standardized application programming interfaces to construct an agent equipped with HBV prevention knowledge. The agent will incorporate identity authentication mechanisms, access control policies, and periodic security audits to ensure user information security. The agent will perform user profiling analysis to extract key characteristics related to HBV prevention, such as HBV knowledge level, risk perception, and screening and vaccination decision status, serving as the basis for tailored interventions. Customized prompts and external tools will be employed by the agent to guide the large language model in generating targeted responses across the three key functions: (i) human-agent interactive consultation, (ii) intelligent case management, and (iii) care coordination.

In the human–agent interactive consultation, users can consult the agent on HBV prevention topics, including transmission routes, screening methods, vaccination schedules, and family-based control measures. Tailored responses will be generated, supporting both text and voice input/output to ensure accessibility for inclusion health populations with varying literacy levels.

In the intelligent case management, the agent will function as a personal health manager with a focus on supporting inclusion health populations in overcoming the barriers to HBV screening and vaccination. It will (i) conduct user profiling through questionnaire-based assessments to identify HBV prevention knowledge gaps, health beliefs, and access barriers to HBV screening and vaccination, and deliver tailored information to address these challenges; (ii) assess users’ decision-making status regarding HBV screening and vaccination, providing tailored counseling, educational videos, and service scheduling links to support informed decision-making; and (iii) provide full-cycle management for HBV screening and vaccination via automated text or voice reminders with follow-up consultations for users who delay or miss scheduled appointments to ensure scheduling completion.

Finally, the agent will collaborate with a nurse-led multidisciplinary team comprising nurses, hepatologists, and public health physicians. Upon receiving abnormal HBV screening results, the agent will classify users for their infection or immunity status and generate tailored recommendations, such as vaccination advice for non-immune individuals or further testing for suspected occult HBV infections. Simultaneously, the agent will notify the nursing team through a backend interface, enabling timely case referral, diagnosis confirmation, new infection reporting, immunization management, and treatment coordination.

Delphi expert consultations will be performed to further refine the development process. A diverse panel of approximately ten experts, comprising engineers specializing in large language models, academic researchers in artificial intelligence, and nursing experts, will be invited to assess the construction phase of the agent. Initial evaluation items will be generated based on the agent construction framework to assess its completeness, standardization, and the scientific validity and feasibility. Evaluations will be conducted using a 4-point Likert scale (1 = disagree, 2 = somewhat agree, 3 = agree, and 4 = strongly agree). Comments on potential improvements or revisions will also be solicited. Items receiving ratings below 3 or those accompanied by expert comments will undergo further revision. The consultation rounds will continue until consensus is achieved, which is defined as agreement by at least 75% of the panel members agree (score of 3 or 4) on an item [47].

### 3.5. Deliver Phase

This phase aims to evaluate the performance of the nurse-led, large language model-empowered agent employed to improve HBV screening and vaccination rates among inclusion health populations through quantitative and qualitative evaluations. The results will be used to iteratively optimize the agent and the intervention delivery among inclusion health populations through collaborative efforts between researchers, specialists, participants, and engineers.

*Quantitative evaluation*. Based on previous studies by the study team [3,40], HBV public health forums, and clinical liver disease expert consultations, 40 testing questions were formulated (Supplementary Material S2, Table S4), covering HBV, its symptoms, transmission routes, screening, vaccination, household isolation, and accidental exposure management. These questions will be posted to the agent and the original model for comparison. Model responses will be documented and assessed independently by two blinded hepatology specialists from two university-affiliated hospitals. To ensure the reliability of the human ratings, inter-rater reliability, including the Cohen’s kappa, will be calculated and any discrepancies will be resolved through consensus discussion.

A framework assessing the quality of information, understanding and reasoning, expression style and persona, safety and harm, and trust and confidence, referred to by the acronym QUEST, will be used to guide the evaluation [48]. First, the quality of information assesses accuracy and relevance of the model responses. To establish a clear ground truth, standard answers will be developed based on current guideline recommendations for HBV prevention, such as HBV screening tests and vaccination schedules. The accuracy of model responses will be determined by their alignment with these gold-standard answers. The relevance of the responses will be assessed by aligning them with the intent of the posted questions. The accuracy and relevance will be further quantified using automated metrics, including the F1 score, calculated by comparing the model responses against the gold-standard references. Second, the understanding and logical reasoning capabilities of the models will be evaluated to assess the extent to which the model accurately interprets the meaning of the input question and whether the generated response demonstrates sound reasoning. A 4-point rating scale will be used to score both comprehension and reasoning quality, with higher scores reflecting stronger performance. Third, the expression style and persona focus on the readability of model responses, which will be evaluated using the Chinese readability objective evaluation formula proposed by Cheng et al. [49]. It focuses on seven core factors, such as the proportion of conjunctions, sematic richness, and sentence length variation, that influence Chinese reading difficulty. A higher score indicates greater reading difficulty. Fourth, the assessment of safety and harm involves calculating the percentages of model responses that contain bias (language that discriminates against inclusion health populations), harm (misinformation that negatively impacts user decisions or behaviors toward HBV prevention), and fabricated information. Finally, trust and confidence aim to evaluate confidence in the models’ ability to generate accurate, fair and safe responses. A 4-point rating scale will be used, with higher scores indicating greater trustworthiness. Differences in these metrics between the agent and the original model will be compared using the Wilcoxon signed-rank test for continuous variables and Chi-square test for categorical variables.

*Qualitative evaluation*. The study will be tested in a randomized controlled pilot study to identify feasibility issues, refine solutions, and explore potential intervention benefits for participants’ HBV screening and vaccination behaviors. The eligible participants are inclusion health individuals who had not undergone HBV screening or vaccination. Based on Whitehead’s recommendations for the required pilot trial sample size [50], 40 participants will be recruited from two university-affiliated hospitals and their community health centers. Participants will be randomly allocated to the control group or intervention group in a 1:1 ratio using a computer-generated block randomization list prepared by an independent statistician. Allocation concealment will be ensured using sequentially numbered, opaque, and sealed envelopes. Two research assistants will enroll participants and reveal the group assignments by opening the envelopes in sequential order. Participants in the control group will receive a standard 15 min oral health education session focusing on HBV prevention, covering the importance of HBV prevention, screening procedures, vaccination schedules, and follow-up care after HBV diagnosis. Participants in the intervention group will be managed using the nurse-led, large language model-empowered agent (Table 1). Individual, in-depth, semi-structured interviews will be conducted at 1 and 6 months with participants in the intervention group. Interviews will specifically focus on exploring the overall user experience, potential technical barriers encountered during interaction with the agent, changes in their knowledge, attitudes, and behaviors related to HBV screening and vaccination, and perceived areas for improvement. Open-ended questions will be asked to encourage discussion, including the following: (i) How do you perceive the program overall? (ii) How do you feel about your interactions with the agent? (iii) What changes in HBV prevention occurred after participation in the program? and (iv) What do you suggest to improve the program? Each interview will be audio-recorded and transcribed verbatim. Thematic analysis will be used to analyze the data. The feedback from inclusion health populations will be used to refine the study. HBV screening and vaccination rates (specifically tracking one-dose, two-dose, and three-dose completion) will be assessed at the six-month follow-up. To ensure consistent data capture across both groups, these outcomes will be verified via electronic medical records. Additionally, secondary feasibility endpoints, including participant recruitment, intervention uptake, and retention rates, will be calculated. Group differences in these outcomes will be evaluated using the Chi-square test.

### 3.6. Ethical Considerations

The study will be conducted in accordance with the principles of the Declaration of Helsinki. Ethical approval was obtained from the institutional ethics committees. Participants will be fully informed about the study aim, their rights of participation and withdrawal, and the assurance of confidentiality. Written consent will be obtained from all participants. For inclusion health individuals who are illiterate, a witness will be present to ensure their comprehension of the study information. A thumbprint will be obtained in lieu of a written signature, with the witness also providing their signature to verify the consent process. All the data will be anonymously and securely stored by the principal researcher and will be destroyed five years after the study completion.

## 4. Discussion

To the best of our knowledge, the study is the first to integrate a large language model-empowered agent into nurse-led care to enhance HBV prevention in inclusion health populations. If the study demonstrates feasibility and yields positive outcomes, it will contribute to HBV diagnosis, immunity, and specialist care linkages, thereby advancing HBV micro-elimination in the most vulnerable cohort. It will also establish a codesign framework for developing domain-specific large language models in nursing care, expanding nurses’ roles in promoting preventive health equity for inclusion health populations.

First, this study will enhance HBV screening and vaccination rates, contributing to HBV micro-elimination in inclusion health populations. In 2016, the World Health Organization adopted the goal of eliminating HBV globally by 2030, defined as a 90% reduction in incidence and a 65% reduction in mortality [51]. Studies from Brazil [52], the United States [53], and France [54] have implemented HBV preventive measures among inclusion health populations, including shelter residents, low-income migrants, and female sex workers. However, even with the provision of free on-site screening and vaccinations, the completion rates for HBV screening and three-dose vaccination have consistently remained below 60% [3]. Significant gaps persist in thoroughly addressing and incorporating healthcare access disparities into program designs. Within the current study, the define phase systematically identified 51 factors influencing HBV screening and vaccination among inclusion health populations. These factors are crucial for comprehensively identifying modifiable intervention targets, including health knowledge, health beliefs, and barriers to healthcare access. Additionally, a qualitative study will be undertaken to further explore the specific cultural barriers to HBV screening and vaccination in the Chinese context, thereby informing culturally tailored strategies. By focusing on these key modifiable factors, this study aims to yield a greater intervention effect on HBV screening and vaccination rates in inclusion health populations. This will ultimately contribute to improved HBV diagnosis, immunity, and care linkage, thereby advancing the micro-elimination goal within this vulnerable cohort.

Second, this study will establish a codesign framework for developing domain-specific large language models in nursing care, paving the way for future innovative nursing services. Although large language models and their empowered agents have demonstrated preliminary positive effects in improving patient health knowledge, self-management behaviors, and emotional and social wellbeing [16], their practical application in the nursing field remains scarce. Several reviews and perspectives have discussed how large language models can support nursing practice, education, and research, but often lack empirical validation [41]. A few studies have tested the feasibility of integrating general-purpose large language models into clinical nursing care; however, they have revealed significant risks of hallucination, misinformation, and biases [55]. A robust methodological framework guiding the refinement of large language models, specifically in the nursing domain, is imperative. The current study addresses this critical requirement by employing established strategies, including fine-tuning and retrieval-augmented generation [46], to adapt to a domain-specific model. This will equip nurses to undertake HBV prevention-related health education, counseling, decision support, and case management. Notably, this study employs the double diamond model to guide the codesign process, fostering extensive collaborations among diverse stakeholders and ensuring a comprehensive understanding of the unique needs of inclusion health populations. The success of this study will help establish a codesign framework that harnesses the transformative potential of large language models within the nursing domain, leading to more effective, equitable, and personalized nursing care.

Third, the innovative service will expand nurses’ roles in addressing preventive health inequities among inclusion health populations within the Chinese context. Health disparities have been widely studied in Western countries, such as the United States [2]. Many frameworks, including the National Institute on Minority Health and Health Disparities research framework, have been developed to understand social determinants of health and guide the development of targeted interventions to mitigate these disparities [3]. Nurses are pivotal in this endeavor, with their practice domains ranging from disease prevention to screening, early detection, and management of various health conditions such as cancers and cardiovascular diseases [11]. However, in China, although population-based studies have highlighted health disparities among certain underprivileged groups (e.g., ethnic minorities, rural migrant workers, and children under five), they often lack an in-depth exploration of the underlying social determinants [56]. There is notable absence of relevant frameworks and interventions designed to address these disparities, and the roles of nurses in this process remain inadequately delineated. The current study, which focuses on HBV prevention, seeks to bridge this gap by comprehensively identifying the determinants of health disparities in HBV prevention through a systematic review [3] and an in-depth qualitative study. It also explicitly outlines nurse-led components, including health education, counseling, decision-making support, and case management, to mitigate these disparities [14]. Importantly, the study ensures equitable access to HBV prevention information for inclusion health populations with diverse healthcare access and health literacy levels by creating a large language model-empowered agent accessible on mobile devices through both text and voice interactions. Hence, with HBV prevention as a pilot program, the study will empower nurses to take a proactive role in promoting preventive health equity, aligning with the Health China Action plan’s goal to achieve health equity by 2030 [56].

Nevertheless, this study is anticipated to encounter several challenges. First, recruiting inclusion health populations within healthcare settings is difficult because financial constraints frequently preclude service utilization. Their limited health literacy may also diminish the perceived importance of HBV prevention and their willingness to participate in the study. To mitigate this, the study will specifically target several subgroups of inclusion health populations that are dominant in the Chinese context, including rural residents and migrant workers. Furthermore, the study settings will encompass two university-affiliated hospitals and their community healthcare settings to broaden the potential participant pool. Second, the follow-up of these populations regarding HBV screening and vaccination utilization will be challenging owing to their unstable employment and living conditions. To address this, the study will implement reminders via the agent and conduct telephone follow-ups by a nurse-led multidisciplinary team to ensure consistent engagement and accurate tracking of service utilization. For future large-scale randomized controlled trials, it is essential to predefine retention tactics and contact protocols, track engagement and contact attempts in both groups, and adopt an intention-to-treat analysis to mitigate potential attrition bias.

This study has several limitations. First, owing to feasibility constraints, this study will not encompass all subgroups of inclusion health populations at a heightened risk of HBV infection, such as female sex workers. Consequently, this study may not fully address the critical needs of all inclusion health populations regarding HBV prevention. Second, the generalizability of this study might be limited due to its focus on China, and cross-cultural adaptation of the content and interface is recommended for replicability in other settings. Third, while the intervention might increase accessibility by reducing the need for face-to-face healthcare access, its dependence on devices and internet connectivity poses a risk of excluding vulnerable subgroups with limited digital access. Finally, it lacks robust assessments of intervention benefits that rely on qualitative interviews. A large randomized controlled trial is essential to empirically assess the intervention effectiveness on the participants’ knowledge, beliefs, and HBV screening and vaccination rates, including the screening completion rates and the three-dose vaccination completion rate. A follow-up duration of 12 months is suggested to evaluate the sustained intervention effectiveness. Most importantly, it should be noted that the intervention group received continuous follow-up whereas the control group received a single session; consequently, any observed differences in outcomes may be driven by the intensity of attention and should be interpreted with caution.

## 5. Conclusions

Based on the Double Diamond model, this study aims to codesign and test a nurse-led, large language model-empowered agent for HBV prevention among inclusion health populations. It will perform human–agent interactive consultation, intelligent case management, and care coordination, as well as collaborate with a nurse-led multidisciplinary team to manage HBV screening, vaccination, and specialist care linkage. The comprehensiveness of the intervention components is ensured by thoroughly exploring modifiable factors influencing HBV screening and vaccination among inclusion health populations and by identifying effective nurse-led intervention components (e.g., health education, counseling, and case management) targeting these factors. Fine-tuning and retrieval-augmented generation techniques will be employed to enhance the performance of agents for HBV prevention. Importantly, the engagement of diverse stakeholders will be pivotal in identifying and addressing the unique HBV prevention needs of inclusion health populations. This study is anticipated to demonstrate feasibility and have positive impacts on HBV screening and vaccination rates among inclusion health populations. It has the potential to advance HBV micro-elimination among this vulnerable cohort while also providing an innovative codesign framework for integrating large language models into nursing practice to address preventive health disparities.

## Figures and Tables

**Table 1:** Overview of the nurse-led, large language model-empowered agent for improving HBV screening and vaccination among inclusion health populations.

Implementers	Intervention Session, Targes, and Components	Goal	Contents	Delivery
Large language model-empowered agent	▪ Session: Human-agent interactive consultation ▪ Targets: Knowledge deficits in HBV prevention ▪ Components: Health education and counseling	▪ To address personalized HBV prevention information needs for inclusion health populations	▪ The agent supports consultations on HBV prevention topics, including transmission routs, screening methods, vaccination schedules, and family-based control measures, generating tailored responses to user queries ▪ Both text and voice input/output will be available to ensure accessibility for inclusion health populations with diverse literacy levels	▪ Time: 1 to 6 months ▪ Format: Individual format ▪ Dose and duration: Need based
	▪ Session: Intelligent case management ▪ Targets: Service access barriers ▪ Components: Health education, counseling, decision support, and case management	▪ To address barriers to HBV screening and vaccination for inclusion health populations through a full-cycle management	▪ Conduct user profiling to assess HBV prevention knowledge, health beliefs, and barriers and provide tailored information to address individual needs (Month 1) ▪ Assess individual decision-making status regarding HBV screening and vaccination and provide tailored decisional support (e.g., tailored counseling and service scheduling links) (Month 2) ▪ Track HBV screening and vaccination status via behavior reminders and follow-up consultations (Month 1 to 6)	▪ Time: 1 to 6 months ▪ Format: Individual format ▪ Dose and duration: Need based. Twice weekly behavior reminders before the scheduling
	▪ Session: Care coordination ▪ Targets: HBV care discontinuity ▪ Components: Care coordination	▪ To coordinate with the nurse-led multidisciplinary team for tailored HBV management	▪ Classify users’ HBV status and generate tailored recommendations, such as further testing for suspected occult HBV infections ▪ Notify the nursing team for prompt referrals, diagnosis, reporting, immunization, and treatment coordination	▪ Time: Upon receiving screening results ▪ Format: Individual format ▪ Dose and duration: Need based
Nurse-led Multidisciplinary Team	▪ Session: HBV screening and vaccination management ▪ Targets: Service access barriers ▪ Components: Counseling	▪ Promote users’ interaction with the agent and address barriers to HBV screening and vaccination	▪ Monitor user interactions with the agent and conduct follow-ups to assess users’ HBV prevention knowledge and offer question and answering support (month 1) ▪ Track HBV screening and three-dose vaccination progress, and initiate consultations to address barriers for users not completing the schedules by the due date (month 1 to 6)	▪ Time: 1 to 6 months ▪ Format: Individual format via telephone ▪ Dose and duration: 1 to 5 doses based on individual need, 15 min/dose
	▪ Session: Specialist HBV care linkage ▪ Targets: HBV care discontinuity ▪ Components: Care coordination and referral	▪ To link specialist HBV care based on screening results	▪ Develop and initiate plans for examination, treatment, and follow-up care based on HBV screening results ▪ Report new HBV infection cases ▪ Assist with specialist care referrals for confirmed cases	▪ Time: Upon receiving screening results ▪ Format: Individual format ▪ Dose and duration: Need based

Abbreviations: HBV, hepatitis B virus.

## Data Availability

Data sharing is not applicable to this study protocol as no new data were collected.

## Supplementary Materials

The following supporting information can be downloaded at: https://www.mdpi.com/article/10.3390/nursrep16020074/s1; S1: SPIRIT 2025 checklist of items to address in a randomized trial protocol; S2: Table S1: Search sources for hepatitis B prevention and management guidelines; Table S2: Search keywords; Table S3: Examples of tailored information for improving HBV knowledge, health beliefs, and healthcare access; Table S4: Testing questions related to hepatitis B and its prevention.

## Public Involvement Statement

Inclusion health populations, engineers, and hepatology specialists will be invited.

## Guidelines and Standards Statement

The study was reported following the SPIRIT (Standard Protocol Items: Recommendations for the Intervention Trials) statement for protocols. A complete list of reporting guidelines can be accessed via the equator network: https://www.equator-network.org/ (accessed on 13 November 2025).

## Abbreviations

The following abbreviations are used in this manuscript:

HBV	Hepatitis B virus
LoRA	Low-rank adaptation
QLoRA	Quantized low-rank adaptation
LLM	Large language model
