# Supplementary material for: Codesigning a Nurse-Led, Large Language Model-Empowered Agent to Increase Hepatitis B Screening and Vaccination for Inclusion Health Populations: A Research Protocol
Source: Nurs Rep. 2026 Feb 19;16(2):74. doi: 10.3390/nursrep16020074 (PMC12942929; doi:10.3390/nursrep16020074)
Supplement: Supplementary file 1 [file nursrep-16-00074-s001.zip › S2.pdf]

Supplementary File S2

Table S1 Search sources for hepatitis B prevention and management guidelines

Categories	Detailed sources
Computerized decision systems	✓ BMJ Best Practice<br>✓ Up to Date
Guideline websites	✓ The World Health Organization<br>✓ The Guidelines International Network<br>✓ The National Institute for Health and Care Excellence<br>✓ The Medlive
Liver disease professional association websites	✓ The China liver health<br>✓ The Asian Pacific Association for the Study of the Liver<br>✓ The European Association for the Study of the Liver<br>✓ The American Association for the Study of Liver Diseases
Electronic databases	✓ Web of Science Core Collection,<br>✓ OVID Medline<br>✓ Embase<br>✓ Scopus<br>✓ Cochrane Library<br>✓ Proquest Health & Medicine Collection<br>✓ EBSCOhost Academic Search Premier<br>✓ EBSCOhost CINAHL Complete<br>✓ Sinomed<br>✓ China National Knowledge Infrastructure

Table S2 Search keywords

Concept	Search Terms
**# Hepatitis B**	Hepatitis B virus* or hepatitis B or HBV or hep B or CHB or hb virus or HBsAg or hepatitis or liver inflammation or hepatic inflammation or liver cirrhosis or liver fibrosis or hepatic cirrhosis or hepatic fibrosis or hepatocellular carcinoma or liver cell carcinoma or liver cancer or (hepatocellular or liver or hepatic cell) adj3 (cancer or carcinoma* or tumor* or tumour* or oncolog*)
**# Guideline**	Guideline* or consens* or standard* or summary statement* or best practice*or evidence* or evidence-based practice* or evidence-based summar* or evidence-based guideline* or evidence summar* or evidence synthes*

Table S3 Examples of tailored information for improving HBV knowledge, health beliefs, and healthcare access

Intervention Targets	Measurements	Tailoring Algorithms	Tailored Information with Messaging Techniques
**HBV knowledge**	An HBV knowledge questionnaire	↪ Correct answer (1 point): affirm the response and ask further questions to reinforce understanding.<br>↪ Incorrect or unsure answer (0 point): provide information to correct the misperception.	For an example item: Will eating with someone who has hepatitis B lead to infection?<br>↪ Response "No": You believe that eating with someone who has hepatitis B will not lead to infection (*descriptive feedback*); your judgment is correct (*evaluative feedback*). Do you think there is a risk of infection if you share cups, chopsticks, or spoons with a hepatitis B patient (*content matching*)?<br>↪ Response "Yes": You believe that eating with someone who has hepatitis B can lead to infection (*descriptive feedback*). This misunderstanding is also prevalent among the public (*comparative feedback*) and is an important cause of discrimination against those with hepatitis B. In fact, hepatitis B is not transmitted through digestive system, so eating with a hepatitis B patient or sharing cups and utensils will not lead to infection *(content matching*).
**Health beliefs about HBV prevention**	An instrument will be validated to measure:<br>↪ Perceived susceptibility of HBV<br>↪ Perceived severity of HBV<br>↪ Perceived benefits of HBV screening<br>↪ Perceived benefits of HBV vaccination	↪ Lower perception (subscale score ≤ 3): use health information and personal story telling to enhance the perception.<br>↪ Higher perception (subscale score > 3): affirm participant responses and ask questions to promote reflections on specific benefits.	For the perceived benefits of HBV screening:<br>↪ Lower perception: The score you assessed for the perceived benefits of HBV screening is_____ (*descriptive feedback*); you seem to disagree with the benefits of HBV screening and may not realize its importance (evaluative feedback). HBV infection usually has no obvious symptoms, and without screening, it can be difficult to know if one has been infected. According to the World Health Organization, there are approximately 300 million hepatitis B patients worldwide, and only approximately 10.5% of them are aware of their infection status. This means that nearly 90% of hepatitis B patients are unaware of their infection and may have progressed to the late stages by the time they seek medical help (*content matching*). Story narrative "I'am 56 years old and have never felt unwell until I couldn't eat and went to the hospital for an examination, where I was diagnosed with hepatitis B cirrhosis and ascites. Before this, I had no idea that I had hepatitis B. I thought that as long as I could eat and work, I wasn't sick, and going for tests would only cost me money…If I could have discovered and treated hepatitis B earlier, it wouldn't have progressed to the middle or late stages (content matching)."<br>↪ Higher perception: The score you assessed for the perceived benefits of HBV screening is ____(descriptive feedback); you acknowledge the benefits of HBV screening, and your judgement is reasonable (evaluative feedback). Can you specifically explain what the consequences would be if HBV infection is not diagnosed timely (content matching)?
**Barriers to HBV screening and vaccination**	A questionnaire will be developed to measure:<br>↪ Decision making status regarding HBV screening and vaccination<br>↪ Barriers to	For HBV screening:<br>↪ Decide to undertake: output information focusing on accessible medical support for both negative and positive results.<br>↪ Decide not to undertake or unsure: explore and address specific barriers.	For HBV screening:<br>↪ Decide to undertake: explain measures to take for negative results (retesting for HBV infection window period) and positive results, as well as the available medical support (*content matching*).<br>↪ Decide not to undertake or unsure: Explain the necessity of HBV screening, the screening procedure, benefits, and potential risks (such as false negatives) to promote informed decision-making. Assess and address specific barriers. For example, in response to the concern about "the long wait time for test results," the corresponding information would be: "The qualitative screening for hepatitis B generally takes about 3 hours to produce results, and we will notify you of the results via your mobile phone, which won't take up much of your time (*content matching*)."

	HBV screening and vaccination	For HBV vaccination:<br>- Decide to undertake: explain the vaccination procedure.<br>- Decide not to undertake or unsure: explore and address specific barriers.	For HBV vaccination:<br>- Decide to undertake: Focus on the vaccination procedure, contradictions, adverse reactions, and vaccination schedule (*content matching*).<br>- Decide not to undertake or unsure: Inform about the basic principles, necessity, effectiveness, and potential risks of HBV vaccination to promote informed decision-making. Assess and address specific barriers. For example, in response to concerns about "the cost of HBV vaccination is high," the information provide would be "The HBV vaccination generally requires 3 doses. There are both domestic and imported vaccines; the domestic vaccine costs about approximately 20 Yuan per dose, while the imported vaccine costs about 100 Yuan per dose. Vaccination can effectively prevent HBV infection and avoid the occurrence of liver cirrhosis and liver cancer." Compared to the cost of receiving the vaccine, preventing HBV can mitigate much greater medical expenses and burdens (*Content matching*)."

Table S4 Testing questions related to hepatitis B and its prevention

No.	Questions
**Category One: Hepatitis B, Its symptom and Disease Course**	
1	What is a hepatitis B virus surface antigen carrier?
2	What is the difference between large and small “San Yang”?
3	What dose large “San Yang” represent?
4	Can hepatitis B virus carriers naturally turn negative?
5	Can chronic hepatitis B be cured?
6	Can someone feel healthy and still be infected with hepatitis B?
**Hepatitis B Transmission Routes**	
7	If my girlfriend is a hepatitis B virus carrier, will I get infected?
8	Can I get infected with hepatitis B by eating together?
9	If I have not been exposed to risk factors, why would I be infected with hepatitis B virus?
10	Can a hepatitis B-infected mother breastfeed a baby who has received the hepatitis B vaccine?
**Hepatitis B Vaccination and Immunity**	
11	Can allergic individuals receive the hepatitis B vaccine?
12	After completing hepatitis B vaccination, how long does immunity last?
13	Can the hepatitis B vaccine be administered alongside other vaccines?
14	Is it safe to receive the hepatitis B vaccine without undergoing screening?
15	What should I do if antibodies are not produced after receiving the hepatitis B vaccine?
16	What should I do if I miss the scheduled time for hepatitis B vaccination?
17	If improving personal and environmental hygiene can prevent disease, is vaccination unnecessary?
18	What is high-titer hepatitis B immunoglobulin, and what is its function?
**Screening and Diagnosis**	
19	How can I know if I have chronic hepatitis B infection?
20	Who should undergo hepatitis B screening?
21	How are chronic hepatitis B test results typically differentiated?
22	Is blood testing the only way to determine if I am infected with hepatitis B?
**Family Isolation, Exposure Management, and Daily Protection**	
23	How should I handle accidental contact with the blood and bodily fluids of a hepatitis B virus infected individual?
24	If one spouse is a hepatitis B virus carrier, do we need to take protective measures during sexual intercourse?
25	How should family members and close contacts of a hepatitis B patient prevent infection?
26	What should other family members do if there is a hepatitis B surface antigen carrier at home?
27	My husband is a hepatitis B patient, and my test shows I have antibodies, but weakly positive; do I still need to get another vaccination?
**Hepatitis B-Related Laboratory Tests and Treatments**	
28	What are ALT and AST transaminases?
29	How should the “two pairs and half” be understood in the diagnosis of hepatitis B?
30	What laboratory indicators should raise concerns about the possibility of liver cirrhosis?
31	What is meant by “the five items of hepatitis B” and “two pairs and half”?
32	If the hepatitis B surface antigen is negative and the hepatitis B surface antibody is positive, what dose this indicate?

33	How can I know if a hepatitis patient's condition is worsening and needs emergency medical attention?
**Impacts of Hepatitis B virus infection**	
34	Can someone with hepatitis B obtain a health certificate?
35	Can the five serological indicators for hepatitis B be tested during school enrollment or employment health check?
36	What impacts do hepatitis B patient face in their daily lives?
37	Dose being infected with hepatitis B affect normal marriage and childbirth?
38	I am a women with hepatitis B; can I get pregnant and have a baby?
39	If I am infected with hepatitis B, should I tell my family?
40	The hepatitis B virus does not hinder finding a partner; if it's time to get married, get married; if it's time to have children, have children?
